# New quinoxalinone inhibitors targeting secreted phospholipase A2 and α-glucosidase

**DOI:** 10.1080/14756366.2017.1363743

**Published:** 2017-08-31

**Authors:** Fatmah A. S. Alasmary, Fatima S. Alnahdi, Abir Ben Bacha, Amr M. El-Araby, Nadine Moubayed, Ahmed M. Alafeefy, Moustafa E. El-Araby

**Affiliations:** a Chemistry Department, College of Science, King Saud University, Riyadh, Saudi Arabia;; b Biochemistry Department, College of Science, King Saud University, Riyadh, Saudi Arabia;; c Faculty of Pharmacy, Ain Shams University, Cairo, Egypt;; d Botany and Microbiology Department, College of Science, King Saud University, Riyadh, Saudi Arabia;; e Department of Chemistry, Kulliyyah of Pharmacy, International Islamic University Malaysia, Kuantan, Malaysia;; f Department of Pharmaceutical Chemistry, Faculty of Pharmacy, King Abdulaziz University, Jeddah, Saudi Arabia;; g Department of Pharmaceutical Organic Chemistry, Faculty of Pharmacy, Helwan University, Cairo, Egypt

**Keywords:** Quinoxalinone, coronary heart disease, diabetic complications, phospholipase A2, α-amylase, α-glucosidase

## Abstract

Elevated blood glucose and increased activities of secreted phospholipase A2 (sPLA2) are strongly linked to coronary heart disease. In this report, our goal was to develop small heterocyclic compound that inhibit sPLA2. The title compounds were also tested against α-glucosidase and α-amylase. This array of enzymes was selected due to their implication in blood glucose regulation and diabetic cardiovascular complications. Therefore, two distinct series of quinoxalinone derivatives were synthesised; 3-[*N*′-(substituted-benzylidene)-hydrazino]-1*H*-quinoxalin-2-ones **3a–f** and 1-(substituted-phenyl)-5*H*-[1,2,4]triazolo[4,3-a]quinoxalin-4-ones **4a–f**. Four compounds showed promising enzyme inhibitory effect, compounds **3f** and **4b–d** potently inhibited the catalytic activities of all of the studied proinflammatory sPLA2. Compound **3e** inhibited α-glucosidase (IC_50_ = 9.99 ± 0.18 µM); which is comparable to quercetin (IC_50_ = 9.93 ± 0.66 µM), a known inhibitor of this enzyme. Unfortunately, all compounds showed weak activity against α-amylase (IC_50_ > 200 µM). Structure-based molecular modelling tools were utilised to rationalise the SAR compared to co-crystal structures with sPLA2-GX as well as α-glucosidase. This report introduces novel compounds with dual activities on biochemically unrelated enzymes mutually involved in diabetes and its complications.

## Graphical abstract

**Figure:**
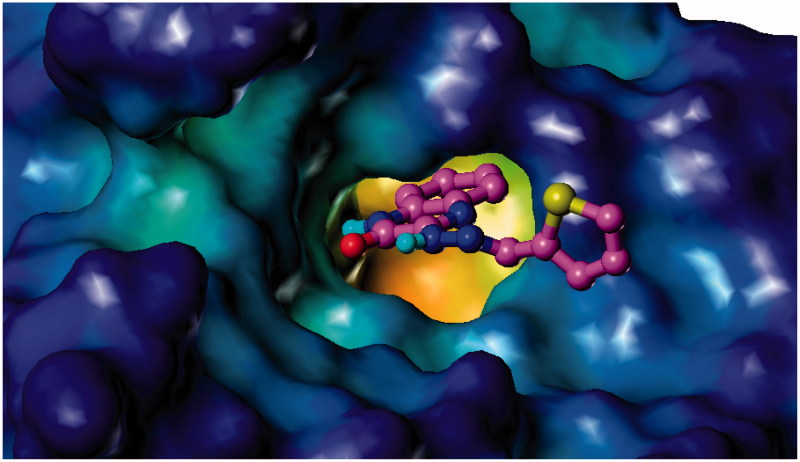

## 1. Introduction

Impaired function of blood vessels and heart tissues is a common health problem in diabetic patients^1^. The individuals with poor control of their blood glucose suffer from dysregulation of lipid metabolism leading to deposition of fats in blood vessels^2^. Therefore, a new consensus has been created about the underlying lipid metabolic markers in coronary heart disease (CHD)^3^. In this regard, phospholipase A2 isozymes have been a focus for many studies as novel markers of CHD. The super family phospholipase A2 is a hydrolase type of enzyme that specifically releases a carboxylic acid from *sn*2 position of glycerophospholipids. The subfamily secretory phospholipase A2 (sPLA2) is common in almost all tissues of the mammalian bodies. The hydrolytic activity of sPLA2s causes the release of poly unsaturated fatty acid arachidonic acid (AA) to surrounding tissues. AA is the precursor of the inflammatory mediators prostaglandins and leukotrienes^4^. Dysregulation of sPLA2 function leads to uncontrolled generation of AA in affected tissues leading to inflammatory disorders such as sepsis^5^, asthma^6^, Alzheimer’s diseases^7^, atherosclerosis^8^ and many others. Chronic elevation of blood glucose leads to increased activation of sPLA2s, generation of AA and eicosanoids and inflammatory CHD.

Several sPLA2 isozymes have been studied for their implication in blood glucose regulation through insulin dependent mechanisms. These enzymes are also involved in cardiovascular complications of diabetes^9^. For example, sPLA2-GIB promotes insulin resistance and hyperglycaemia in transgenic mice overexpressing this enzyme^10^. Group X sPLA2 is another regulator of insulin production, where it suppresses glucose stimulated insulin secretion from beta cells through a COX-2 dependent mechanism^11^. Diabetic subjects were found to be more susceptible to lipolysis by group V sPLA2 increasing their state of inflammation and enhancing their risk of cardiovascular complications^12^. In the light of their inflammatory and diabetogenic activities, inhibition of secretory phospholipases is hypothesised to be of value in ameliorating the inflammatory and cardiovascular complications of diabetes. A very unique characteristic of secretory phospholipases is their requirement of calcium as a cofactor in high (millimolar) concentrations. This property makes them amenable for inhibition via ligands bearing metal chelating groups. Many of the previously reported inhibitors of sPLA2 contain a hydrophobic moiety and a metal chelating group either linked directly or appended to a central heterocyclic scaffold^13–15^.

Diabetic patients suffer from an elevated blood glucose level that is mediated by a number of interplaying enzymes and biomechanisms. Of the major contributors to elevated post-prandial blood glucose are carbohydrate hydrolases including α-glucosidase and α-amylase^16^. These enzymes hydrolyse polysaccharides leading to the availability of monomeric sugars for absorption, and thus the elevation of post-prandial blood glucose level^17^. The inhibition of such enzymes is therefore of merit for the modulation of post-prandial blood glucose^16^ ^,^ ^18^. Many inhibitors have been reported for such enzymes and are marketed for their antihyperglycemic effect^19^.

In this work, we are reporting novel heterocyclic sPLA2 inhibitors based on a quinoxalinone scaffold. The synthesised inhibitors were assayed for their activity against sPLA2 GIB, IIA, V, X, and XII. To expand our investigation of our compounds’ activities, we also investigated their ability to inhibit α-glucosidase and α-amylase to assess their potential in controlling post-prandial blood glucose^16^ ^,^ ^20^ ^,^ ^21^. The inhibition results against the carbohydrate hydrolases were compared to quercetin, a flavonoid well known to inhibit α-glucosidase and α-amylase^22–24^. We hypothesise that simultaneous inhibition of this group of interrelated biological targets may serve as a starting point for a novel small molecule approach in controlling hyperglycaemia and cardiovascular complications concomitantly in diabetic patients. This is due to the ability of these compounds to not only inhibit proatherogenic sPLA2 targets, but also due to their ability to control hyperglycaemia which acts as their inducer.

## 2. Experimental

### 2.1. Chemistry

The melting points (mp) were determined using a Gallenkamp melting point apparatus. The IR spectra were recorded in KBr discs on a PerkinElmer FT-IR (Spectrum BX) spectrophotometer (*ν*_max_ in cm^−1^). ^1^H and ^13^C NMR spectra were recorded using a JEOL Resonance spectrometer (500 MHz “megahertz”). Coupling constants are given in Hertz (Hz). Deuterated DMSO-d_6_ solvent was obtained from Goss Scientific Instruments (Cheshire, UK). DMSO-d_6_ was stored in silica gel desiccators. Elemental analysis was carried out by the microanalysis service using 2 mg of the sample (Micro Analytical Center, Faculty of Science, Cairo University). Spectral data IR, NMR and Elemental analysis confirmed the structures of the compounds. The purity of all of the compounds (>95%) was established by elemental analyses (C, H, N) and results were within 0.4% of the calculated values. All of the known compounds were identified by comparing their analytical and physicochemical data with previously reported data. The NMR data for the compounds are provided in the Supplementary Information.

### 2.2. General procedure A to the synthesis of 3-[*N*′-(substituted-benzylidene)-hydrazino]-1H-quinoxalin-2-one (3a–f)

3-[*N*′-(substituted-benzylidene)-hydrazino]-1*H*-quinoxalin-2-one (**3a–f**) have been prepared according to Shiho and Tagami^30^ and Rashed et al.^31^, a mixture of 3-hydrazino-1*H*-quinoxalin-2-one^2^ and the corresponding aromatic aldehyde (0.01 mol of each) in ethanol as solvent (20 ml) was refluxed for 5 h. Upon cooling, the precipitate was obtained, filtered, dried and purified by column chromatography (8:1.5:0.5 dichloromethane/ethylacetate/ethanol) to give the desired product.

### 2.3. General procedure B to the synthesis of 1-(substituted-phenyl)-5H-[1,2,4]triazolo[4,3-a]quinoxalin-4-ones (4a–f)

1-(Substituted-phenyl)-5*H*-[1,2,4]triazolo[4,3-a]quinoxalin-4-ones **4a–f** have been prepared according to Cheeseman^32^, mixture of **3a–f** (0.02 mol) and anhydrous sodium acetate (0.04 mol) was stirred in glacial acetic acid (100 ml), then 10% v/v solution of bromine in glacial acetic acid (10 ml) was added in a small portion. The reaction mixtures were stirred at 25 °C for 1 h, and then poured onto ice water (200 ml). The precipitates were filtered washed with water, followed by aqueous NaHSO_3_ solution (10%) (2 × 50 ml). Finally, dried and purified by column chromatography (8:1.5:0.5 dichloromethane/ethylacetate/ethanol) to give the desired product.

#### 2.3.1. (E)-3-(2-(3,4,5-trimethoxybenzylidene)hydrazinyl)quinoxalin-2(1H)-one (3a)

Prepared according to general procedure A. Yield 91%, as a yellowish orange solid; mp 258–260 °C, IR (KBr, cm^−1^) *ν*_max_ = 3466 (N–H), 3096 (C–H, *sp2*), 1671 (C=O), 1566 and 1498 (C=N). ^1^H NMR (500 MHz, DMSO-d_6_) *δ* (ppm): 12.4 (s, 1 H, NHCO), 11.19 (s, 1 H, NH), 8.47 (s, 1 H, CH), 7.52 (s, 1 H, H-6′), 7.50 (s, 1H, H-2′), 7.18 (dd, 2H, *J* = 11.80 Hz, 2.8 Hz, H-6 and H-7), 6.98 (d, 2H, *J* = 12 Hz, H-5 and H-7), 3.37 (s, 3H, 3′-OCH_3_), 3.71 (s, 3H, 4′-OCH_3_), 3.84 (s, 3H, 5′-OCH_3_). ^13^C NMR (500 MHz, DMSO-d_6_) *δ* (ppm): 157.3, 154.9, 153.2, 150.8, 146.8, 146.1, 145.2, 138.4, 130.3, 128.7, 125.4, 123.5, 115.1, 106.1, 104.1, 60.1, 56.1, and 55.9. Anal. calcd for C_18_H_18_N_4_O_4_=C, 61.01; H, 5.12; N, 15.81; found, C, 61.10; H, 4.85; N, 16.01.

#### 2.3.2. (E)-3-(2-(2,4-dinitrobenzylidene)hydrazinyl)quinoxalin-2(1H)-one (3b)

Prepared according to general procedure A. Yield 98%, as a red solid; mp 283–285 °C, IR (KBr, cm^−1^) *ν*_max_ = 3287 (N–H), 3056 (C–H, *sp2*), 1673 (C=O), 1568 and 1522 (C=N). ^1^H NMR (500 MHz, DMSO-d_6_) *δ* (ppm): 12.4 (s, 1H, NHCO), 12.00 (s, 1H, NH), 9.06 (s, 1H, CH), 8.76 (dd, 1H, *J=* 15 Hz, 2.5 Hz, H-5′), 8.55 (dd, 1H, *J* = 6.00 Hz, 2.5 Hz, H-6′), 8.42 (s, 1H, H-3′), 7.54 (d, 2H, *J* = 7.00 Hz, H-5 and H-8), 7.22 (m, 2H, H-6 and H-7), ^13^C NMR (500 MHz, DMSO-d_6_) *δ* (ppm): 150.6, 147.2, 146.7, 146.1, 139.2, 134.8, 132.2, 129.2, 128.9, 127.4, 126.0, 125.7, 123.6, 120.4, and 115.7. Anal. calcd for C_15_H_10_N_6_O_5_=C, 50.85; H, 2.85; N, 23.72; found; C, 50.54; H, 2.56; N, 23.66.

#### 2.3.3. (E)-3-(2-(2,4-dihydroxybenzylidene)hydrazinyl)quinoxalin-2(1H)-one (3c)

Prepared according to general procedure A. Yield 85%, as a yellow solid, mp 292–294 °C, IR (KBr, cm^−1^) *ν*_max_ = 3330 (N–H), 3050 (C–H, *sp2*), 1686 (C=O), 1558 and 1418 (C=N). ^1^H NMR (500 MHz, DMSO-d_6_) *δ* (ppm): 12.36 (s, 1H, NHCO), 11.95 (s, 1H, NH), 11.46 (s, 1H, OH); 9.93 (s, 1H, OH), 8.58 (s, 1H, CH), 7.40 (d,1H, *J* = 3.00 Hz, H-3′), 7.19 (m, 4H, H-5 and H-6 and H-7 and H-8), 6.53 (dd, 2H, *J=* 10.00 Hz, 2.5 Hz, H-5′ and H-6′), ^13^C NMR (500 MHz, DMSO-d_6_) *δ* (ppm): 160.3, 159.5, 150.7, 148.4, 145.8, 132.8, 131.4, 128.7, 125.4, 124.6, 123.5, 115.1, 110.9, 107.6, and 102.8. Anal. calcd for C_15_H_12_N_4_O_4!_=C, 57.27; H, 3.81; N, 17.81; found; C, 57.62; H, 3.45; N, 18.02.

#### 2.3.4. (E)-3-(2-(3,4-dichlorobenzylidene)hydrazinyl)quinoxalin-2(1H)-one (3d)

Prepared according to general procedure A. Yield 67%, as a yellow solid, mp 244–246 °C, IR (KBr, cm^−1^) *ν*_max_ = 3438 (N–H), 3042 (C–H, *sp2*), 1682 (C=O), 1568 and 1522 (C=N). ^1^H NMR (500 MHz, DMSO-d_6_) *δ* (ppm): 12.42 (s, 1H, NHCO). 11.39 (s, 1H, NH), 8.52 (s, 1H, CH); 8.28 (m, 2H, H-5′ and H-6′), 7.90 (m, 1H, H-2′), 7.20 (m, 4H, H-5 and H-6 and H-7 and H-8), ^13^C NMR (500 MHz, DMSO-d_6_) *δ* (ppm): 172.0, 155.0, 150.7, 146.1, 143.8, 135.6, 131.7, 131.0, 127.9, 115.1, and 115.0. Anal. calcd for C_15_H_10_Cl_2_N_4_O=C, 54.07; H, 3.03; N, 16.82; found; C, 54.08; H, 2.81; N, 16.94.

#### 2.3.5. (E)-3-(2-(thiophen-2-ylmethylene)hydrazinyl)quinoxalin-2(1H)-one (3e)

Prepared according to general procedure A. Yield 66%, as a yellow solid, mp 260–262 °C, IR (KBr, cm^−1^) *ν*_max_ = 3271 (N–H), 3042 (C–H, *sp2*), 1673 (C=O), 1573 and 1414 (C=N). ^1^H NMR (500 MHz, DMSO-d_6_) *δ* (ppm): 12.38 (s, 1H, NHCO), 11.20 (s, 1H, NH), 8.74 (s, 1H, CH), 7.61 (d, 1H, *J* = 4.5 Hz, H-2′), 7.35 (d, 1H, *J* = 3.0 Hz, H-3′), 7.19 (d, 1H, *J* = 3.5 Hz, H-4′), 7.11–7.17 (m, 4H, H-5 and H-6 and H-7 and H-8). ^13^C NMR (500 MHz, DMSO-d_6_) *δ* (ppm): 150.8, 145.9, 141.8, 139.5, 132.8, 129.9, 128.6, 128.3, 127.8, 125.4, 124.6, 123.5, and 122.9. Anal. calcd for C_13_H_10_N_4_OS=C, 57.76; H, 3.37; N, 20.73; found; C, 57.63; H, 3.39; N, 20.68.

#### 2.3.6. (E)-3-(2-(3-methoxybenzylidene)hydrazinyl)quinoxalin-2(1H)-one (3f)

Prepared according to general procedure A. Yield 98%, as a yellowish orange solid, mp 283–285 °C, IR (KBr, cm^−1^) *ν*_max_ = 3400 (N–H), 3180 (C–H, *sp2*), 1688 (C=O), 1567 and 1419 (C=N). ^1^H NMR (500 MHz, DMSO-d_6_) *δ* (ppm): 12.4 (s, 1H, NHCO), 11.22 (s, 1H, NH), 8.55 (s, 1H, CH), 7.53 (s, 1H, H-2′), 7.35–7.24 (m, 3H, H-4′ and H-5′ and H-6′), 7.20 (dd, 4H, *J* = 8.25 Hz, 3.5 Hz, H-5 and H-6 and H-7 and H-8), 3.79 (s, 3H, 3′-OCH_3_). ^13^C NMR (500 MHz, DMSO-d_6_) *δ* (ppm): 172.0, 159.5, 150.8, 146.6, 139.2, 134.8, 132.2, 129.2, 128.9, 127.4, 126.0, 125.7, 123.6, 120.4, 115.7, and 55.5. Anal. calcd for C_16_H_14_N_4_O_2_=C, 65.30; H, 4.79; N, 19.04; found; C, 65.17; H, 4.42; N, 18.98.

#### 2.3.7. 1-(3,4,5-Trimethoxyphenyl)-[1,2,4]triazolo[4,3-a]quinoxalin-4(5H)-one (4a)

Prepared according to general procedure B. Yield 95.7%, as a white solid, mp 227–229 °C, IR (KBr, cm^−1^) *ν*_max_ = 3418 (N–H), 3072 (C–H, *sp2*), 1691 (C=O), 1615 and 1419 (C=N). ^1^H NMR (500 MHz, DMSO-d_6_) *δ* (ppm): 7.40 (m, 2H, H-7 and H-8). 7.27 (s, 2H, H-2′ and H-6′). 7.11–7.04 (m, 2H, H-6 and H-9). 3.91 (s, 3H, 3′-OCH_3_), 3.83 (s, 3H, 4′-OCH_3_), 3.78 (s, 3H, 5′-OCH_3_), ^13^C NMR (500 MHz, DMSO-d_6_) *δ* (ppm): 153.9, 152.3, 151.5, 149.5, 145.4, 144.3, 129.6, 128.6, 124.8, 123.9, 120.9, 117.7, 116.1, 112.3, 110.6, 61.6, 60.5, and 56.9. Anal. calcd for C_18_H_16_N_4_O_4_=C, 61.36; H, 4.58; N, 15.90; found; C, 61.73; H, 4.80; N, 15.53.

#### 2.3.8. 1-(2,4-Dinitrophenyl)-[1,2,4]triazolo[4,3-a]quinoxalin-4(5H)-one (4b)

Prepared according to general procedure B. Yield 92%, as brownish orange crystals, mp >300 °C, IR (KBr, cm^−1^) *ν*_max_ = 3418 (N–H), 3083 (C–H, *sp2*), 1690 (C=O), 1533 and 1419 (C=N). ^1^H NMR (500 MHz, DMSO-d_6_) *δ* (ppm): 12.4 (s, 1H, NHCO), 9.08 (s, 1H, H-3′), 8.86 (dd, 1H, *J* = 8.0 Hz, 2.5 Hz, H-5′), 8.23 (d, 1H, *J=* 8.0 Hz, H-6′), 7.43 (dd, 2H, *J* = 8.0 Hz, 2.5 Hz, H-6 and H-9), 6.99 (m, 2H, H-7 and H-8), ^13^C NMR (500 MHz, DMSO-d_6_) *δ* (ppm): 151.5, 149.5, 148.1, 145.3, 144.5, 134.7, 129.3, 129.3, 128.6, 127.9, 123.4, 121.2, 120.1, 117.3, and 116.2. Anal. calcd for C_15_H_8_N_6_O_6_=C, 51.14; H, 2.29; N, 23.86; found; C, 51.26; H, 2.29; N, 23.99.

#### 2.3.9. 1-(2,4-Dihydroxyphenyl)-[1,2,4]triazolo[4,3-a]quinoxalin-4(5H)-one (4c)

Prepared according to general procedure B. Yield 95%, as a brown solid, mp >300 °C, IR (KBr, cm^−1^) *ν*_max_ = 3403 (N–H), 3062 (C–H, *sp2*), 1666 (C=O), 1500 and 1425 (C=N). ^1^H NMR (500 MHz, DMSO-d_6_) *δ* (ppm): 12.13 (s, 1H, NHCO), 7.64 (s, 1H, H-3′), 7.53 (d, 2H, *J* = 10.5 Hz, H-5′ and H-6′), 7.15 (m, 4H, H-6 and -7H and H-8 and H-9), 6.72 (s, 2H, OH). ^13^C NMR (500 MHz, DMSO-d_6_) *δ* (ppm): 151.8, 148.1, 144.3, 132.9, 129.1, 127.8, 124.5, 123.2, 116.9, 115.7, 102.1, and 101.2. Anal. calcd for C_15_H_10_N_4_O_3_=C, 61.22; H, 3.43; N, 19.04; found; C, 61.09; H, 3.63; N, 19.32.

#### 2.3.10. 1-(3,4-Dichlorophenyl)-[1,2,4]triazolo[4,3-a]quinoxalin-4(5H)-one (4d)

Prepared according to general procedure B. Yield 92.5%, as a brownish orange solid, mp >300 °C, IR (KBr, cm^−1^) *ν*_max_ = 3469 (N–H), 3053 (C–H, *sp2*), 1679 (C=O), 1517 and 1412 (C=N). ^1^H NMR (500 MHz, DMSO-d_6_) *δ* (ppm): 12.14 (s, 1H, NHCO), 8.06 (d, 1H, *J* = 2.0 Hz, H-2′), 7.93 (d, 1H, *J* = 8.0 Hz, H-6′), 7.75 (dd, 1H, *J* = 10.0 Hz, 2.0 Hz, H-5′), 7.1–7.4 (m, 4H, H-6 and H-7 and H-8 and H-9); ^13^C NMR (500 MHz, DMSO-d_6_) *δ* (ppm): 152.3, 149.1, 144.9, 132.3, 132.1, 129.9, 128.4, 123.4, 117.7, and 116.9. Anal. calcd for C_18_H_18_N_4_O_4_=C, 54.40; H, 2.44; N, 16.92; found; C, 54.67; H, 2.46; N, 16.55.

#### 2.3.11. 1-(Thiophen-2-yl)-[1,2,4]triazolo[4,3-a]quinoxalin-4(5H)-one (4e)

Prepared according to general procedure B. Yield 39.6%, as a beige solid, mp >300 °C, IR (KBr, cm^−1^) *ν*_max_ = 3467 (N–H), 2923 (C–H, *sp2*), 1687 (C=O), 1517 and 1413 (C=N); ^1^H NMR (500 MHz, DMSO-d_6_) *δ* (ppm): 12.14 (s, 1H, NHCO), 7.50 (m, 1H, H-2′), 7.40 (m, 1H, H-3′), 7.30 (m, 1H, H-4′), 7.16–7.14 (m, 4H, H-6 and H-7 and H-8 and H-9), ^13^C NMR (500 MHz, DMSO-d_6_) *δ* (ppm): 151.6, 144.6, 144.0, 133.2, 131.4, 129.4, 128.5, 128.1, 122.9, 120.7, 117.2, 116.4, and 116.1. Anal. calcd for C_13_H_18_N_4_O_4_=C, 58.20; H, 3.01; N, 20.88; found; C, 58.66; H, 3.68; N, 20.90.

#### 2.3.12. 1-(3-Methoxyphenyl)-[1,2,4]triazolo[4,3-a]quinoxalin-4(5H)-one (4f)

Prepared according to general procedure B. Yield 55%, as a beige solid, mp 286–288 °C, IR (KBr, cm^−1^) *ν*_max_ = 3469 (N–H), 3049 (C–H, *sp2*), 1686 (C=O), 1587 and 1408 (C=N). ^1^H NMR (500 MHz, DMSO-d_6_) *δ* (ppm): 12.18 (s, 1H, NHCO), 8.29 (s, 1H, H-2′), 7.80 (m, 1H, H-6′), 7.38 (m, 1H, H-5′), 7.28–7.25 (m, 4H, H-6 and H-7 and H-8 and H-9), 6.93 (m, 1H, H-4′), 3.82 (s, 3H, 3′-OCH_3_). ^13^C NMR (500 MHz, DMSO-d_6_) *δ* (ppm): 159.5, 151.9, 150.4, 144.2, 130.4, 127.7, 122.6, 121.9, 117.2, 116.8, 116.2, 115.1, and 55.6. Anal. calcd for C_16_H_12_N_4_O_2_=C, 65.75; H, 4.14; N, 19.17; found; C, 65.85; H, 4.40; N, 18.78.

### 2.4. Biology: general methods

#### 2.4.1. Inhibition of sPLA2 activity

The test of inhibitory activity of sPLA2 was performed as described by Lobo de Araujo and Radvanyi^41^. Briefly, the substrate consisted of 3.5 mM lecithin in a mixture of 3 mM NaTDC “Sodium taurodeoxycholate”, 100 mM NaCl, 10 mM CaCl_2_, and 0.055 mM red phenol as colorimetric indicator in 100 ml H_2_O. The pH of the reaction mixture was adjusted to 7.6. The human group IB (pG-IB), IIA (hG-IIA), V (hG-V), X (hG-X), and XII (hG-XII) sPLA2 were solubilised in 10% acetonitrile at a concentration of 0.02 μg/μL. A volume of 10 μL of these PLA2 solutions was incubated with 10 μL of each compound for 20 min at room temperature. Then, 1 ml of the PLA2 substrate was added, and the kinetic of hydrolysis was followed during 5 min by reading the optical density at 558 nm. The inhibition percentage was calculated by comparison with a control experiment (absence of compound).

#### *2.4.2. α-*Glucosidase *inhibitory activity*

The α-glucosidase inhibitory activity was performed by measuring the liberation of 4-nitrophenol as described by Andrade-Cetto et al.^42^. Briefly, 20 μL of control drug (quercetin), DMSO, or studied compound (0.78–12.5 μg/ml) were mixed with 180 μL of the α-glucosidase enzyme from *Saccharomyces cerevisiae* (Sigma, St. Louis, MO) and incubated at 37 °C for 2 min. Then, 150 μL NPGP (4-nitrophenyl β-d-glucopyranoside) were added and the samples were incubated at 37 °C for about 20 min. The assay media contained 10 mM of potassium phosphate buffer, pH 6.9, 5 mM of 4-NPGP, and 2 U of α-glucosidase. Quercetin also was used as positive control at the same concentration of the tested compound while DMSO was used as the negative control. A microplate reader was used for samples reading at 405 nm. The inhibition percentage was calculated using the following equation: 100 – (X2 sample − X1 sample/X2 control − X1 control) × 100 [where X1 is the absorbance of the initial reading (*T*_0_), X2 is absorbance of the final reading (*T* = 15 min)]. IC_50_ values were determined by nonlinear regression.

#### 2.4.3. α-Amylase inhibitory activity

The α-amylase inhibitory activity was determined calorimetrically according to Subramanian et al.^43^. Briefly, 4 U of α-amylase enzyme (Sigma, St. Louis, MO) was incubated with 10 μL of each compound (250–31.2 μg/ml), DMSO (negative control), or quercetin (control drug used at the same compound concentrations), at 37 °C for 5 min. Then, samples were mixed with 180 μL of the Amylase Substrate (Labtest^®^) and incubated for 10 min and the first reaction was measured at 620 nm. Then, 100 μL of the reactive α-amylase (Labtest^®^) diluted in distilled water (1:1) and 150 μL of distilled water were added in the microplate and then incubated at 37 °C for 10 min and the second reaction was measured again. IC_50_ values were determined by nonlinear regression. The α-amylase inhibition percentage was determined using the following equation: % inhibition =100 – (A2 sample – X1 sample/X2 control − X1 control) × 100 where X1 is the absorbance of the initial reading and X2 is the absorbance of the final reading.

### 2.5. Molecular modelling

Molecular modelling was performed using Sybyl-X program. The crystal structures of hG-X sPLA2 and α-glucosidase were downloaded from the RCSB website (www.rcsb.com) (PDB IDs: 5G3M and 3W37 respectively). Protein preparation was performed using the Biopolymer Preparation tool according to the following parameters: H-Addition, H-Bond; Termini treatment, charged; Protonation type of histidines, according to H-bonding donor or acceptor; Side chain Bumps, Fix by Lovell method. At the end of the preparation, brief Staged Energy Minimisation was performed to the amino acid residues only using the following parameters: Iterations, 100; Initial Minimisation, None; Force Field, MMFF94s for α-glucosidase and AMBER7 FF02 for hG-X sPLA2; Charges, MMFF94 α-glucosidase and for; Dielectric constant, Constant; Non-Bonding Cutoff, 8.0 Å. The 3D structures were generated by Concord protocol of Sybyl-X and saved as SLN files to be used for docking. Docking was performed using the Dock Ligands protocol of Sybyl-X using Surflex Docker. The protomol was generated using the co-crystallised ligand. The docked ligands were then inspected to compare the similarities and differences in binding modes with the co-crystallised ligands.

## 3. Results and discussion

### 3.1. Chemistry

Previously, we reported a small set of quinoxalinone derivatives as antibacterial agents^25^. Therefore, we envisioned the quinoxalinone nucleus as a common pharmacophore to develop compounds that simultaneously inhibit sPLA2 and carbohydrate hydrolases. The compounds incorporate triazole or hydrazine–Schiff base moieties which could coordinate calcium and could therefore serve as a good starting point for sPLA2 inhibition^26^ ^,^ ^27^. Several novel substituted phenyl and thienyl derivatives were synthesised to explore the compound SAR.

Several routes are available for synthesis of quinoxaline ring, depending on the starting nuclei, to give either substituted quinoxaline or fused quinoxaline with other heterocyclic moieties such as, triazole.

**Scheme 1.**
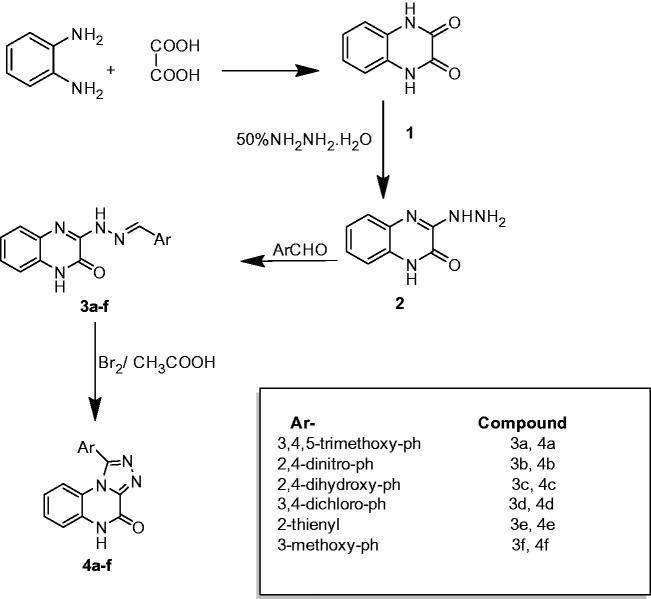
2,3-Substituted-quinoxaline and 1,4-substituted-[1,2,4]triazolo[4,3-*a*]quinoxalin derivatives.

**Figure 1.**
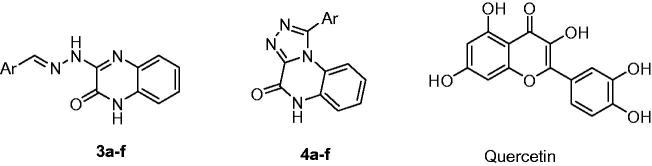
Chemical structures of series 3, series 4, and quercetin.

In our previous work, we have synthesised some quinoxaline derivatives as shown in Scheme 1 starting from 1,4-dihydro-quinoxaline-2,3-dione **1** via the reaction between *o*-phenylenediamine and oxalic acid^28^, then the later compound was used to prepare 3-hydrazino-1*H*-quinoxalin-2-one **2** after reaction with hydrazine hydrate as reported in the literature^29^. In the present work, compound **2** was refluxed with derivatives of aromatic benzaldehyde to afford six novel derivatives of 3-[*N*′-(substituted-benzylidene)-hydrazino]-1*H*-quinoxalin-2-one **3a–f** ^30^ ^,^ ^31^, which then submitted the later compounds to the cyclisation method in the presence of bromine in acetic acid, to give another six novel derivatives of 1-(substituted-phenyl)-5*H*-[1,2,4]triazolo[4,3-a]quinoxalin-4-ones **4a–f** ^32^ (see Figure 1 for the synthesised derivatives).

### 3.2. Inhibition of phospholipase A2 (sPLA2), α-amylase, and α-glucosidase

In order to evaluate the potential anti-inflammatory activity of the 12 studied compounds, we tested their inhibitory effects using several human sPLA2 isozymes, pG-IIA, hG-V, hG-X, and hG-XII, involved in the inflammatory process and porcine pG-IB sPLA_2_ which hydrolyses dietary phospholipids.

Data recorded in Table 1 show that compounds **3f**, **4b**, **4c**, and **4d** have the most promising results in inhibiting the catalytic activities of all studied proinflammatory hG-IIA, hG-V, hG-X, and GXII sPLA2. All of the studied compounds showed low micromolar IC_50_ values against the different sPLA2 isozymes (IC_50_ < 20 µM), while having considerable selectivity towards the proinflammatory sPLA2 isozymes over the pG-IB sPLA2 (Table 1). This may allow future optimisation of the compounds to achieve absolute selective activity towards proinflammatory sPLA2 isozymes. Some of the compounds showed very potent submicromolar IC_50_ values against proinflammatory sPLA2. For instance, compounds **3f**, **4b**, **4c**, and **4d** exhibited potent activities against sPLA2 isozymes hG-IIA, hG-V, hG-X, and hG-XII (IC_50_ 0.33–3 µM). However, the compounds showed lower activity against pG-IB (IC_50_ = 9.31 µM for compound **3a**) (Table 1).

**Table 1.** Inhibitory activity of the title compounds against isoforms of sPLA2 expressed as IC_50_ (µM)±standard deviation.

	IC_50_ (µM) Against Phospholipase				
Compound	pG-IB	hG-IIA	hG-V	hG-X	hG-XIIA
**3a**	9.31 ± 0.36	2.48 ± 0.28	1.46 ± 0.47	1.26 ± 0.05	3.04 ± 0.67
**3b**	10.72 ± 0.67	5.36 ± 0.73	1.12 ± 0.31	2.54 ± 0.42	2.28 ± 0.08
**3c**	13.33 ± 0.50	3.71 ± 0.27	9.45 ± 0.13	12.48 ± 0.87	6.75 ± 0.17
**3d**	13.50 ± 1.02	4.50 ± 0.33	5.70 ± 0.27	3.00 ± 0.63	10.50 ± 1.74
**3e**	10.72 ± 0.92	2.81 ± 0.33	6.28 ± 0.40	4.43 ± 1.03	3.81 ± 0.99
**3f**	12.40 ± 0.03	1.90 ± 0.30	1.22 ± 0.30	0.33 ± 0.06	0.84 ± 0.07
**4a**	14.19 ± 1.22	13.05 ± 0.05	11.30 ± 0.08	12.20 ± 0.47	13.25 ± 0.21
**4b**	13.62 ± 0.70	0.76 ± 0.01	1.53 ± 0.14	1.05 ± 0.08	2.58 ± 0.43
**4c**	14.78 ± 0.44	1.32 ± 0.16	1.05 ± 0.13	0.61 ± 0.03	0.91 ± 0.05
**4d**	14.49 ± 0.87	1.81 ± 0.04	3.04 ± 0.57	2.71 ± 0.07	1.41 ± 0.07
**4e**	16.77 ± 0.93	16.81 ± 0.41	18.63 ± 0.56	17.89 ± 0.94	18.63 ± 1.33
**4f**	15.97 ± 1.71	17.10 ± 0.41	15.80 ± 0.85	14.36 ± 0.52	15.77 ± 1.29
Oleanolic acid	10.40 ± 1.16	11.50 ± 0.76	16.42 ± 1.53	16.531 ± 1.38	13.14 ± 0.92

All experiments were performed in duplicate.

Several reports on transgenic mice that overexpress group IIA, group V, and group X sPLA2 have all shown an increase in foam cell formation and atherosclerosis which has been reduced through the use of an inhibitor of sPLA2 activity^33–35^. Thus, the potential use of sPLA2 inhibitors for preventing many diseases like cardiovascular events is being investigated which may provide optimal benefit for these patients.

Concerning the pancreatic α-glucosidase enzyme (Table 2), the studied compounds showed reasonable activities with satisfactory IC_50_ values (IC_50_ < 50 µM) (Table 2). Compound **3e** inhibited the enzyme and showed an IC_50_ value (9.99 ± 0.18 µM) comparable to that recorded for quercetin (9.3 ± 0.66 µM). Several studies reported that some foods and herbs have potential beneficial effects on diabetic glycaemic control by inhibiting these enzymes^36^ ^,^ ^37^. Hence, retardation of starch digestion by inhibition of digestive enzymes plays a key role in the control of diabetes and metabolic syndrome. The α-amylase activity of the studied compounds was irrelevant (IC_50_ > 200 µM) (Table 2). Compound **3e** showed IC_50_ value of 395.84 ± 35.88 making it practically inactive against α-amylase. The compounds were therefore unable to inhibit α-amylase despite their promising activity against α-glucosidase and sPLA2.

**Table 2.** Inhibitory activity of the title compounds against α-glucosidase and α-amylase expressed as IC_50_ (µM) ± standard deviation.

	IC_50_ (µM)	
Compound	α-Glucosidase	α-Amylase
**3a**	42.33 ± 3.38	428.93 ± 31.04
**3b**	14.11 ± 2.08	296.38 ± 26.81
**3c**	14.18 ± 1.51	300.38 ± 26.32
**3d**	22.51 ± 2.01	735.36 ± 52.52
**3e**	9.99 ± 0.18	395.84 ± 35.88
**3f**	13.93 ± 2.03	492.68 ± 46.20
**4a**	14.75 ± 2.12	638.56 ± 30.65
**4b**	22.99 ± 2.83	806.19 ± 91.97
**4c**	12.91 ± 1.69	1087.43 ± 74.08
**4d**	12.98 ± 1.20	431.81 ± 34.72
**4e**	36.52 ± 2.98	1330.65 ± 82
**4f**	24.29 ± 3.76	413.95 ± 52.68
Quercetin	9.93 ± 0.66	479.75 ± 18.86

All experiments were performed in duplicate.

### 3.3. Molecular modelling

**Figure 2.**
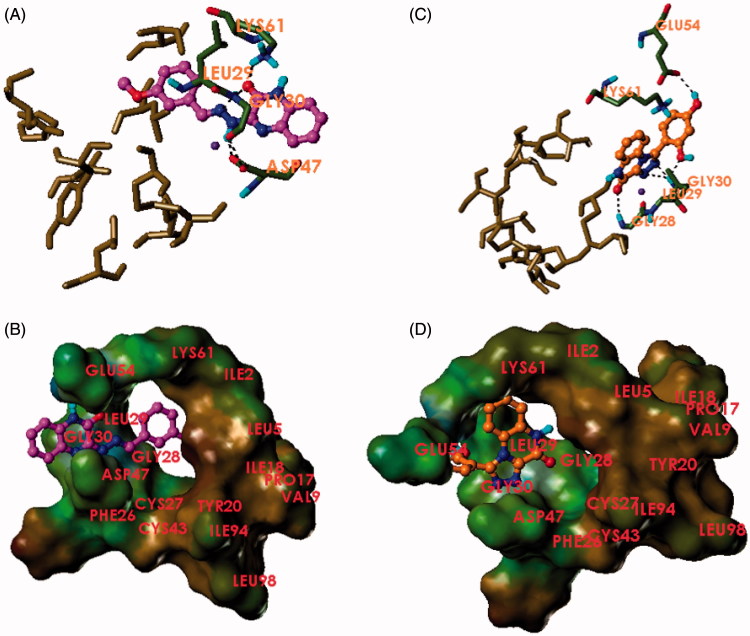
Binding mode of **3f** and **4c** to hG-X sPLA2. (A) Compound **3f** (magenta) and (C) compound **4c** (orange) in the binding pocket of hG-X sPLA2 showing hydrogen bonds, calcium coordination (purple), and interacting residues. (B) Compound **3f** (magenta) and (D) compound **4c** (orange) in the binding pocket of hG-X sPLA2 showing a lipophilic potential surface of the pocket.

To investigate the binding mode of the synthesised inhibitors to different enzyme targets, molecular modelling techniques were deployed using the co-crystal structure of hG-X sPLA2 with the hit compound **1** reported by Giordanetto et al.^38^. In this co-crystal structure, the 4-benzylphenyl moiety of the inhibitor occupies a hydrophobic pocket, while the amide group coordinates with the catalytic site calcium. Manual docking of the compound **3f** in the active site of the crystal structure of hG-X sPLA2 (PDB ID: 5G3M) shows the fulfilment of key interactions for the inhibition of this enzyme. The hydrazine nitrogen forms a critical coordination bond with the calcium ion present as a cofactor for the enzyme, similar to that of the amide moiety of the co-crystallised inhibitor. This interaction plays a central role in the inhibition of PLA2 activity^38^. The 3-methoxyphenyl moiety of **3f** occupies the same hydrophobic pocket occupied by the 4-benzylphenyl moiety of the co-crystallised inhibitor. This pocket is composed of Ile2, Leu5, Val9, Leu 29, Ile94, Leu98, Tyr20, Cys43, Cys27, Pro17, and Ile18. The dramatic decrease in activity in **3c** (IC_50_ = 12.48 ± 0.87 µM) is proposed to be due to the lack of hydrophobic substituents on the phenyl ring and their replacement with two hydrophilic 2,4-dihydroxy groups. This causes an obvious reduction in hydrophobic interaction with this pocket, forcing a hydrophilic moiety into a pocket hydrophobic in nature. This conclusion is in accordance with the activities of the rest of series 3, since the compounds bearing hydrophobic groups show potent activities (<5 µM) varying according to the nature of the substituent and their steric volumes. It is worthy to note that the nitro groups of compound **3b** do not impart hydrophilicity to the molecule, but rather “hydroneutrality” as shown by Sagawa and Shikata^39^. Compound **3e**, which bears an unsubstituted 2-thienyl moiety, possesses the least activity following **3c** due to the low extent of hydrophobic interactions. The quinoxalinone nucleus is found to penetrate a tunnel formed by Leu29 and Lys61 beyond the metal pocket. The carbonyl of the quinoxalinone moiety is able to form two hydrogen bonds with Lys61 and Gly30 anchoring the compound in position. Another hydrogen bond is also found between the hydrazine nitrogen and the carboxylic acid group of Asp47 (Figure 2).

The docking of compound **4c** in the active site of hG-X sPLA2 shows a binding mode astonishingly different from series **3**. The compounds do a 180° turn to put the quinoxalinone moiety in the direction of the hydrophobic pocket. Hydrophobic interactions are limited to Ile2, Leu29 and the side chain of Lys61 and the penetration of the hydrophobic pocket is very shallow. The carbonyl oxygen forms a hydrogen bond with the nitrogen of Gly28. The triazole moiety forms a monodentate coordination bond with calcium, and hydrogen bonding with the nitrogen of Gly30. This metal coordination is a strong interaction that is essential for fixation of the compound and achieving inhibition. The 2,4-dihydroxyphenyl moiety is found to penetrate the Leu29-Lys61 tunnel and establish two hydrogen bonds with the nitrogen of Gly30 and the carboxylic group of Glu54. Due to the superficial penetration of the hydrophobic pocket and very limited hydrophobic interactions, the activity of the compounds is governed by the extent of hydrogen bonding, where the derivatives bearing hydrophilic and/or hydrogen bonding groups (**4b** and **4c**) or able to form halogen bonds (**4d**) exhibit superior activities compared to those lacking such groups (**4a**, **4e**, and **4f**). Thus, hG-X sPLA2 inhibition by series **4** is a function of the crucial metal coordination and the extent of hydrogen bonding with the enzyme, but not due to hydrophobic contact, which is rather very poor in this case.

**Figure 3.**
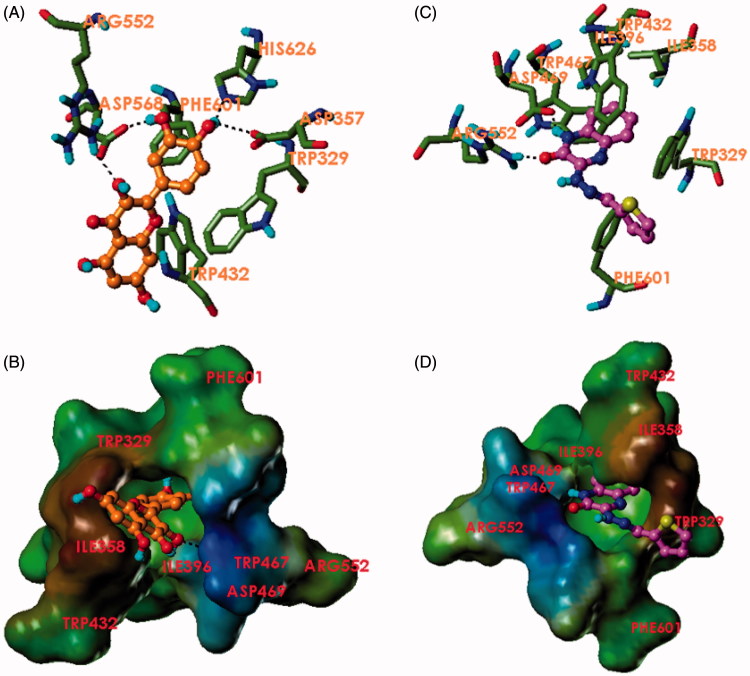
Binding mode of quercetin and **3e** to α-glucosidase. (A) Quercetin (orange) and (C) compound **3e** (magenta) in the binding pocket of α-glucosidase showing hydrogen bonds and interacting residues. (B) Quercetin (orange) and (D) compound **3e** (magenta) in the binding pocket of α-glucosidase showing a lipophilic potential surface of the pocket.

Binding of the compound **3e** to the active site of α-glucosidase was investigated via molecular docking and compared to that of quercetin using crystal structure of α-glucosidase co-crystallised with the inhibitor acarbose (PDB ID: 3W37)^40^. The docking results showed very comparable docking poses of the two ligands. Both compounds were able to achieve hydrogen bonding with Arg552, similar to that found in the co-crystal structure. Quercetin was able to achieve three other hydrogen bonding interactions with the carboxylic group of Asp568 and Asp357 and the imidazole of His626 while **3e** formed another hydrogen bond with Asp469 via its amidic quinoxalinone nitrogen. Interestingly, both quercetin and **3e** formed an impressive number of hydrophobic interactions. Compound **3e** was shown to insert its quinoxalinone benzene ring into a hydrophobic pocket composed of Ile358, Ile396, Trp467, Trp329, and Trp432. The benzene ring of the quinoxalinone nucleus was able to form a T-shaped π–π stacking with the indole ring of Trp329. The compound is bent in an L-shape to achieve another T-shaped π–π stacking with Trp329 through its thiophene ring. The thiophene also forms hydrophobic contact with the phenyl of Phe601. Quercetin achieved a T-shaped π–π stacking with another tryptophan residue, Trp432. The binding of quercetin also showed hydrophobic contacts with Phe601 and Trp329. This finding is in accordance with the report by Li et al. where the binding of quercetin to α-glucosidase was shown to be governed by hydrophobic interaction through a kinetic study^23^. Compound **3e** also shows comparable activity to quercetin and a variety of hydrophobic interactions. Compound **3a** showing the least potency did not form any hydrogen bonds with the enzyme, but only hydrophobic interactions with Trp467 and Trp565 and a T-shaped π–π stacking with Trp329. This may be due to the steric effects of the 3 adjacent methoxy groups (Figure 3).

## 4. Conclusions

The aim of this work was to synthesise quinoxaline derivatives and to evaluate them *in vitro* as inhibitors against secretory phospholipase A2 (sPLA2) and test their activity against carbohydrate hydrolases (α-glucosidase and α-amylase). Twelve compounds were synthesised based on the 2,3-quinoxaline and triazoloquinoxaline derivatives, where 11 compounds were novel. Regarding the inhibition of proinflammatory sPLA2 isozymes, promising and potent results were recorded for compounds **3f**, **4b**, **4c**, and **4d**, while exhibiting considerably lower activities against GI-B sPLA2. Compound **3e** was the most active compound inhibiting the α-glucosidase with potency comparable to that of the control quercetin. Compound **3e** also showed irrelevant activity towards α-amylase. The mechanism of action of the compounds was investigated through molecular modelling and correlated to the practically obtained results. Despite the absence of activity against α-amylase, our compounds were able to dually inhibit α-glucosidase and sPLA2 isozymes. This work opens a new area for developing of small molecules that could simultaneously modulate postprandial blood glucose and inhibit proatherogenic sPLA2 isozymes with the aim of controlling hyperglycaemia and cardiovascular complications in diabetic patients.
